# Identification of bacterial sRNA regulatory targets using ribosome profiling

**DOI:** 10.1093/nar/gkv1158

**Published:** 2015-11-05

**Authors:** Jing Wang, William Rennie, Chaochun Liu, Charles S. Carmack, Karine Prévost, Marie-Pier Caron, Eric Massé, Ye Ding, Joseph T. Wade

**Affiliations:** 1 Wadsworth Center, New York State Department of Health, Albany, NY 12208, USA; 2 RNA Group, Department of Biochemistry, University of Sherbrooke, Sherbrooke, Quebec, J1H 5N4, Canada; 3 Department of Biomedical Sciences, University at Albany, Albany, NY 12201, USA

## Abstract

Bacteria express large numbers of non-coding, regulatory RNAs known as ‘small RNAs’ (sRNAs). sRNAs typically regulate expression of multiple target messenger RNAs (mRNAs) through base-pairing interactions. sRNA:mRNA base-pairing often results in altered mRNA stability and/or altered translation initiation. Computational identification of sRNA targets is challenging due to the requirement for only short regions of base-pairing that can accommodate mismatches. Experimental approaches have been applied to identify sRNA targets on a genomic scale, but these focus only on those targets regulated at the level of mRNA stability. Here, we utilize ribosome profiling (Ribo-seq) to experimentally identify regulatory targets of the *Escherichia coli* sRNA RyhB. We not only validate a majority of known RyhB targets using the Ribo-seq approach, but also discover many novel ones. We further confirm regulation of a selection of known and novel targets using targeted reporter assays. By mutating nucleotides in the mRNA of a newly discovered target, we demonstrate direct regulation of this target by RyhB. Moreover, we show that Ribo-seq distinguishes between mRNAs regulated at the level of RNA stability and those regulated at the level of translation. Thus, Ribo-seq represents a powerful approach for genome-scale identification of sRNA targets.

## INTRODUCTION

RNAs represent a major class of regulatory molecule in bacteria. ‘Small RNAs’ (sRNAs) are typically non-coding RNAs, 50–150 nt in length (1). Most sRNAs function by interacting with target mRNAs through complementary base pairing, although some sRNAs are known to directly interact with proteins. sRNA:mRNA interaction can positively or negatively impact gene expression at the level of translation initiation, mRNA stability or transcription termination (1). The majority of characterized sRNA:mRNA interactions involve the mRNA 5′ UTR, and affect mRNA stability and/or translation initiation. Repression of translation typically occurs due to occlusion of the Shine-Dalgarno (S-D) sequence and/or start codon as a result of sRNA binding. Activation of translation typically occurs due to secondary structure alterations around the S-D/start codon as a result of sRNA binding to an upstream region on the transcript.

Many sRNA:mRNA base-pairing interactions are facilitated by the RNA chaperone Hfq. Moreover, many sRNAs are stabilized by their association with Hfq. Structural studies of Hfq have identified two distinct RNA-binding surfaces, each with a different sequence preference: the ‘proximal’ face of the Hfq hexamer binds U-rich sequences in sRNAs, such as those derived from intrinsic transcription terminators (2); the ‘distal’ face of the Hfq hexamer binds A-R-N sequences in mRNAs (3,4). In addition to stabilizing sRNAs and facilitating sRNA:mRNA interaction, Hfq promotes degradation of many mRNAs hybridized to an sRNA, due to an interaction between Hfq and RNase E. Hfq association with sRNA:mRNA hybrids often results in RNase E-dependent degradation of both the mRNA and the sRNA (5). Positive or negative effects of sRNAs on mRNA stability can also be due to regulation of translation initiation, since untranslated mRNAs are more prone to degradation (1).

*Escherichia coli* RyhB is one of the best-studied sRNAs. RyhB has ≈50 known target genes and has been shown to regulate many of the corresponding mRNAs directly (i.e. base-pairing interactions have been experimentally demonstrated) (6,7). The majority of RyhB target genes are associated with iron utilization (6,7). Under conditions of iron limitation, RyhB represses expression of many non-essential genes for which the corresponding proteins bind iron. Thus, RyhB has a critical ‘iron-sparing’ function under iron-limiting conditions. RyhB regulates its target genes using a wide variety of mechanisms, all of which involve changes in translation initiation and/or mRNA stability (8). The majority of RyhB target genes are repressed (7), although there are two translationally activated genes, *shiA* and *cirA* (9,10).

Computational prediction of targets is an important problem for diverse classes of regulatory RNAs, and has been most extensively applied to metazoan microRNAs (11). For bacterial sRNAs, this is more challenging than microRNA target prediction for a number of reasons: (i) base-paired regions are fairly short; (ii) base-pairing can involve multiple discontinuous regions of the sRNA or mRNA; (iii) sRNAs are far longer than the region involved in base-pairing so there is a high degree of uncertainty about the location of interaction; indeed, different regions of the same sRNA can base-pair with different mRNA targets (1); (iv) sRNA secondary structure can influence sRNA:mRNA hybridization. Nonetheless, there are several proposed features of sRNA:mRNA interactions that can improve bioinformatic prediction. First, structured regions of sRNAs and mRNAs are typically prevented from pairing, as they are not directly accessible for hybridization (12,13). Second, sequence conservation of both the sRNA and mRNA tends to be higher at the regions of base-pairing (13,14). Third, pairing typically requires a ≈7 bp block of continuous base-pairing at one end of the paired region (15,16). Fourth, an ‘A-R-N’ motif facilitates Hfq binding to the mRNA near the putative site of the sRNA:mRNA hybridization, substantially increasing the likelihood of hybridization (12). However, Hfq binding sites are not universally required for regulation by sRNAs. Notably, many sRNAs do not require Hfq for their function. Furthermore, many species lack Hfq, although analogous proteins have been identified even in species that have Hfq homologues (17,18). sRNA target prediction tools have been developed that incorporate many of these features (14,19–23). These methods are helpful for the identification of targets for numerous sRNAs, including RyhB; however, they are associated with large numbers of false positives and false negatives (14,19).

Genome-scale identification of sRNA targets using experimental approaches has also proved challenging. Transcription profiling using microarrays has been used to identify targets of several sRNAs (24), including RyhB (7). A major weakness of transcription profiling for identification of sRNA targets is the inability to detect effects on translation. Ribosome profiling (Ribo-seq) is a recently developed genome-scale approach that can simultaneously determine RNA levels and levels of translation *in vivo* (25). To date, the use of Ribo-seq has been rather limited in bacterial systems (26–32). While one sRNA has been studied using Ribo-seq (32), data from this study do not allow for a comprehensive assessment of Ribo-seq as a tool for identifying sRNA targets because the sRNA in question appears to have only one target gene. Here, we use Ribo-seq to comprehensively validate known, and identify numerous novel regulatory targets of *E.coli* RyhB. Our data show that Ribo-seq is a powerful approach for experimental identification of sRNA targets, and can reveal sRNA regulation at the level of mRNA stability and at the level of translation.

## MATERIALS AND METHODS

### Strains and plasmids

All strains and plasmids used in this work are listed in Supplementary Table S1. All oligonucleotides used in this work are listed in Supplementary Table S2.

Strains JXW026, JXW028 and JXW029 were constructed using the FRUIT recombineering system (33). Oligonucleotides JW4984 and JW4985 were used to amplify *thyA* from pAMD001. The polymerase chain reaction (PCR) products were electroporated into AMD054 containing pKD46, to replace *ryhB* with *thyA*, yielding JXW026. The pair of oligonucleotides JW5707 and JW5708 contained the *ryhB* mutation for strain JXW029. The pair of oligonucleotides JW5367 and JW5368 contained the *ryhB* mutation for strain JXW028. Each of these pairs, together with JW4986 and JW4887 were used to amplify PCR products containing *ryhB* mutations and flanking sequences, using a colony of MG1655 as template, with SOEing PCR (34). These PCR products were electroporated into JXW026 containing pKD46, to replace *thyA* with each of the two mutant *ryhB* genes. The native *thyA* locus was replaced as previously described (33), yielding JXW028 or JXW029.

Strains MPC144 and KP1184 are derivatives of KP1174 (*rne131 zce*-726::Tn*10*, Hfq^3xFLAG^, Δ*araB*::*kan*, Δ*ryhB*::*cat*). The *rne131* allele has been described previously (5). Construction of *ryhB* (Δ*ryhB*::*cat*) and *araB* (Δ*araB*::*kan*) deletions was described previously (6,9). To construct chromosomal *hfq*^3xFlag^, we used the method described in (35). A Flippase recognition target (FRT)-flanked kanamycin resistance cassette was generated by a 2 step-PCR from the pKD4 plasmid (36) using oligonucleotides EM1689 and EM1690 in the first PCR and oligonucleotides EM1691 and EM1690 in the second PCR. The resulting PCR product was transformed into DY330 after induction of λRed according to (37), selecting for kanamycin resistance. Following P1 transduction of *hfq*^3xFlag^::*kan*, the kanamycin resistance cassette was removed using Flippase (Flp)-encoding helper plasmid pCP20, as described (36).

All *lacZ* reporter gene fusions were constructed in plasmid pAMD-BA-*lacZ* as described previously (38) using the oligonucleotides listed in Supplementary Table S2. For translational fusions pJXW009–030, the upstream region of each gene was amplified using the oligonucleotides JW5072–5115, as indicated in Supplementary Table S2, using a colony of MG1655 as template. To clone pJXW032, the *katG* gene and surrounding sequence were amplified by PCR with JW5086 and JW5175, and cloned into pAMD-BA-*lacZ*. To construct pJXW031 and pJXW033, a strong, constitutive promoter was amplified from pAMD001 using JW3379 and JW3415. The upstream region of *ynfF* (−60 to +24 relative to the translation start) or *fumB* (−77 to +24) was amplified using JW5066 and JW5116 (*ynfF*) or JW5079 and JW5119 (*fumB*). The two PCR products were fused using SOEing (34) and cloned between the *Sph*I and *Hin*dIII restriction sites of pAMD-BA-*lacZ*, using the In-Fusion kit (Clontech). To construct pJXW034–42, the entire 5′ UTR of each gene (as indicated in Supplementary Table S1) was amplified using the oligonucleotides listed in Supplementary Table S2, fused to a constitutive promoter, and cloned between the *Sph*I and *Hin*dIII restriction sites of pAMD-BA-*lacZ*, using the In-Fusion kit (Clontech).

To construct transcriptional fusions pJXW038–040, the upstream sequence together with the entire translated reading frame was amplified using oligonucleotides JW5082 and JW5170, JW5084 and JW5171, JW5100 and JW5172, or JW5110 and JW5173, using a colony of MG1655 as template. The PCR products were cloned between the *Sph*I and *Nhe*I sites of pAMD033 (includes the sequence AATGCATGAAGGAGATATACATATGGC upstream of *lacZ*, with the underlined sequence indicating the translation start codon) (39), using the In-Fusion kit (Clontech).

The mutated *fepB* translational fusion pJXW043 was constructed using PCR products made with oligonucleotides JW5084 and JW5712, JW5085 and JW5711, with a colony of MG1655 as template, using SOEing PCR (34). The resulting fragment was cloned between the *Sph*I and *Hin*dIII sites of pAMD-BA-*lacZ*, using the In-Fusion kit (Clontech). The mutated *cirA* translational fusion pJXW042 was constructed similarly, using oligonucleotides JW5102, JW5382, JW5103 and JW5381.

### Extract preparation

Extracts were prepared as described previously (26), with modifications. 200 ml LB medium was inoculated with 2.0 ml of overnight *E.coli* culture, and grown at 37°C to an OD600 of ≈0.5. Arabinose was added to 0.1%, 10 min before harvesting the cells, to induce the transcription of RyhB. Two minutes prior to harvesting, chloramphenicol was added to a final concentration of 100 μg/ml. Cells were harvested by rapid filtration by using a 500 ml 0.45 μm PES filter system (Celltreat). Cells were flash frozen in liquid nitrogen together with 0.65 ml lysis buffer (26). The frozen cells were pulverized six times at 15 Hz for 3 min by a mixer mill (Retsch MM400). The grinding jars were re-chilled in liquid nitrogen to keep the cells frozen between each cycle. After the pulverized cells were recovered, a small scoopful was saved for bacterial transcript enrichment.

### Transcript enrichment for RNA-seq

The pulverized cell powder was extracted with acid phenol and chloroform followed by isopropanol precipitation as described previously (26). 16S and 23S ribosomal RNAs were removed by subtractive hybridization using Ambion MICROBExpress kit (Life Technologies) following the manufacturer's instructions. The enriched mRNAs were randomly fragmented as described previously (26).

### Ribosome footprinting of mRNA

Footprinting and monosome isolation were performed as described previously (26). Briefly, the pulverized cells were thawed. Clarified lysates were treated with micrococcal nuclease (Worthington Biochemical Corp.). Monosomes were isolated by sucrose gradient fractionation, as judged by agarose gel elecrophoresis of a sample of each fraction. mRNA footprints were purified with acid phenol and chloroform extraction, followed by isopropanol precipitation.

### Library generation and deep sequencing

Both ribosomal footprints and total mRNAs were converted into cDNA libraries as described (26), with modifications. The RNA molecules were dephosphorylated by treating with T4 polynucleotide kinase (New England Biolabs). Polyacrylamide gel purification was performed for size selection of ≈28 nt RNA fragments. Approximately 25–30 nt poly-A tails were added to recovered RNA fragments with an Ambion poly(A) tailing kit (Life Technologies), following the manufacturer's instructions. The polyadenylated RNA samples were reverse transcribed using JW2364 and SuperScript III (Life Technologies), and the reverse transcription products were circularized by CircLigase™ ssDNA ligase (Epicentre). For the footprint libraries, ribosomal RNAs were subtracted from circularized ssDNA using biotinylated sense-strand oligonucleotides JW2808, JW2809, JW2810, and JW2811, as described (26). PCR amplification was performed using the circularized cDNA as template, JW2365 and JW2366 (Table S2) as primers, and Phusion High-Fidelity DNA Polymerase (New England Biolabs). PCR-amplified DNA libraries were deep sequenced using an Illumina HiSeq 2500 instrument (University at Buffalo Next-Generation Sequencing and Analysis Expression Core Facility). Raw data are available through EBI Array Express [Accession number: E-MTAB-2903].

**Table 1.** Putative RyhB targets identified by Ribo-seq

Gene	RNA-seq^a^		Ribo-seq^a^		Notes^b^	References
Repressed Genes	Fold Change	*P*-value	Fold change	*P*-value		
*bfr*	1.26	0.0024	2.68	0.00005	I	(6)
*sodB*	1.55	0.00005	2.44	0.00005	R/P	(7,14,19,23)
*ynfF*	0.24	0.54	2.41	0.0008	P/V	(23)
*dmsA*	0.02	0.95	2.13	0.00005	P/V	(14)
*sufB*	0.66	0.077	2.00	0.0002	I/P	(7,14)
*hscA*	0.76	0.047	1.99	0.00005	R/P	(7,19)
*nrfA*	−0.03	0.94	1.95	0.0070	N/V	This study
*sdhA*	0.98	0.74	1.91	0.00095	R/P	(7,14)
*sdhC*	0.98	0.032	1.80	0.00095	R	(7)
*sdhD*	0.97	0.013	1.80	0.026	R/P	(7,14,19,23)
*fumA*	0.46	0.20	1.76	0.00005	R/P	(7,14)
*acnB*	0.69	0.11	1.75	0.0002	R	(7,58)
*fumB*	0.27	0.48	1.74	0.00055	P/V	(14)
*sdhB*	0.97	0.013	1.73	0.00005	R	(7)
*nuoG*	0.76	0.13	1.68	0.001	R	(7)
*napD*	1.00	0.52	1.68	0.43	N	This study
*iscR*	0.80	0.03	1.67	0.0003	R	(7)
*hypB*	0.33	0.53	1.66	0.0057	P	(14)
*erpA*	−0.05	0.89	1.61	0.0002	P/R	(14)
*msrB*	0.33	0.35	1.57	0.0007	R/P	(7,14)
*napA*	1.19	0.019	1.56	0.0006	N/V	This study
*fhuF*	0.82	0.038	1.52	0.0035	I	(7)
*nuoA*	0.67	0.070	1.47	0.0003	R/P	(7,19)
*nuoC*	0.78	0.048	1.44	0.00065	R	(7)
*fdoG*	0.64	0.14	1.39	0.0016	R	(7)
*mrp*	0.41	0.24	1.38	0.00045	R	(7)
*nuoH*	0.70	0.13	1.37	0.17	R	(7)
*nuoI*	0.61	0.085	1.37	0.0011	R	(7)
*hybA*	0.01	0.99	1.35	0.20	R	(7)
*dhaK*	0.88	0.015	1.34	0.0012	N/V	This study
*frdA*	0.60	0.14	1.34	0.0033	R/P	(7,14,23)
*sufA*	0.61	0.14	1.33	0.0095	I	(7)
*fepB*	0.28	0.46	1.32	0.016	N/V	This study
*napH*	1.10	0.058	1.32	0.31	N	This study
*dhaL*	0.87	0.015	1.31	0.0014	P	(19)
*fdx*	0.71	0.048	1.30	0.002	R	(7)
*yagT*	0.14	0.70	1.29	0.0040	P	(23)
*napF*	0.14	0.84	1.28	0.48	P	(23)
*iscA*	0.78	0.35	1.28	0.69	R	(7)
*fdoH*	0.68	0.28	1.22	0.17	R	(7)
*yhjX*	1.42	0.0036	1.19	0.034	N	This study
*fdoI*	0.62	0.44	1.18	0.33	R	(7)
*hybO*	−0.21	0.56	1.18	0.0042	R	(7)
*katG*	0.58	0.10	1.18	0.004	N/V	This study
*acnA*	0.39	0.27	1.16	0.0049	R/P	(6,7,19)
*napC*	0.39	0.28	1.15	0.022	N	This study
*nagZ*	0.36	0.30	1.14	0.0086	P/R	(14,23)
*fhuA*	0.73	0.047	1.14	0.013	I	(7)
*frdB*	0.81	0.14	1.11	0.0033	R	(7)
*dhaM*	0.85	0.016	1.11	0.007	N	This study
*napB*	0.99	0.058	1.10	0.031	N	This study
*nuoE*	0.79	0.29	1.10	0.083	R	(7)
*nuoB*	0.75	0.043	1.09	0.0088	R	(7)
*ydbK*	0.30	0.41	1.09	0.011	R	(7)
*iscU*	0.74	0.045	1.07	0.016	R	(7)
*ykgJ*	−0.40	0.74	1.07	0.011	P/V	(14)
*narG*	0.13	0.77	1.07	0.022	P	(19)
*yeaC*	0.23	0.53	1.06	0.0095	R	(7)
*gltB*	0.23	0.52	1.06	0.014	N	This study
*ygiQ*	−0.11	0.76	1.04	0.024	P	(14,23)
*iscX*	0.63	0.085	1.04	0.012	R	(7)
*metH*	0.22	0.52	1.01	0.015	P	(14,23)
*iscS*	0.71	0.03	1.00	0.013	R	(7)
*napG*	1.06	0.066	0.98	0.53	N	This study
Activated Genes						
*yegD*	−0.29	0.11	−1.85	0.0015	N/V	This study
*cspB*	−0.86	0.048	−1.41	0.001	N/F	This study
*ugpB*	−0.43	0.27	−1.39	0.0032	N/F	This study
*shiA*	−0.26	0.48	−1.39	0.0022	R/P	(10,19)
*yncE*	−0.60	0.082	−1.38	0.0011	N/V	This study
*cirA*	−0.49	0.18	−1.38	0.0025	P/R/V	(9,19)
*ftnA*	−1.34	0.0013	−1.31	0.0048	I	(6)
*ynaE*	−0.37	0.72	−1.29	0.025	N	This study
*garL*	−0.96	0.025	−1.28	0.017	N	This study
*garP*	−1.20	0.0039	−1.22	0.039	N/F	This study
*flgG*	−0.41	0.35	−1.14	0.038	N	This study
*cpdA*	−0.60	0.16	−1.12	0.014	P	(14)
*yoaK*	−0.44	0.32	−1.07	0.035	N	This study
*garD*	−0.45	0.25	−1.06	0.036	N/F	This study
*ygdQ*	−0.38	0.27	−1.01	0.021	I	(7)
*yjbE*	−1.01	0.026	0.14	0.82	N	This study

^a^Fold-change for RyhB^−^/RyhB^+^ (log_2_). Only genes with changes >2-fold are listed.

^b^R = Reported Previously; P = Predicted, N = Novel; V = Verified in this study; I = reported to be Indirect; F = Failed verification.

### Analysis of deep sequencing data for sRNA target identification

Sequences were aligned to the MG1655 genome using the CLC Genomics Workbench. The number of reads mapping to each gene was determined using custom Python scripts. Differences in expression between pBAD-*ryhB* and pNM12 strains were determined from two independent replicates using the Pathogen Portal ‘RNA-Rocket’ RNA-seq Analysis Pipeline (40) that includes Bowtie (version 2.02, default settings; for aligning reads to reference genomes) (41), Cufflinks (version 2.1.1, default settings; for transcript mapping), and CuffDiff (version 2.1.1, default settings; for comparing expression of transcripts between samples) (42). When the signal level was low for a given gene (number of sequence reads below 200), the gene was excluded from analysis.

### Quantitative reverse transcription PCR

RyhB RNA levels were assessed using reverse transcription coupled with real-time PCR with the oligonucleotides JW3150 and JW3151, using a 7500 fast real-time PCR system (Applied Biosystems) as described previously (43).

### β-galactosidase assays

Overnight cultures were inoculated in 3 ml M9 medium with 0.4% fructose and 30 μg/ml chloramphenicol, and grown to an OD_600_ of 0.3–0.6. β-galactosidase assays were performed as previously described (38). Three independent replicates were performed for all assays. Activity for the first replicate of each RyhB^−^ (MG1655 Δ*lacZ* Δ*ryhB*) sample was assigned a value of 100. All other samples were normalized relative to this value. All assay values are the mean of three independent experiments.

### Prediction of sRNA binding sites on target mRNA

Our new methodology for prediction of sRNA binding sites on a target mRNA is described by the following series of computations:

#### RNA secondary structure prediction

Since the secondary structures of both the target mRNA and the sRNA can play important roles in sRNA:target hybridization, we first made secondary structure prediction for each RNA. To take into account the likelihood that an RNA molecule can exist *in vivo* in dynamic equilibrium among a population of structures, we used the Sfold program (44) for structure prediction, because its algorithm generates a statistically representative sample from the Boltzmann-weighted ensemble of RNA secondary structures (45). A sample size of 1000 structures has been shown to be sufficient for reproducibility of major structural characteristics (46,47). In addition, centroids of structural clusters present representative structures (48).

#### Identification of sRNA:mRNA hybridization regions and prediction of hybrid conformations

Because single-stranded regions, e.g. hairpin loops, can facilitate the nucleation step (i.e. initial intermolecular base pairing) of RNA:RNA interaction, we specifically focused on regions of the sRNA that are predicted to be single-stranded with high probabilities (46). For each such region, the RNAhybrid program (49) was then run to identify potential hybridization regions on the target mRNAs. In the case of RyhB sRNA, we identified a large hairpin loop in the centroid of a major structure cluster. This hairpin starts at C^37^ and ends at U^51^, and is consistent with a proposed structure (50). Since hybridization can elongate beyond the site of nucleation, we included nucleotides upstream and downstream of the hairpin loop. Specifically, we focused on the region from C^28^ to U^68^ for RNAhybrid analysis.

#### Structure-based energetic computation

Given a region with hybrid conformation predicted by RNAhybrid, we further performed structure-based computation for several energetic parameters to model the hybridization process. Δ*G*_T-disruption_ is the target disruption energy, which represents the free energy cost to unpair the target's intramolecular base pairs at the site of hybridization. To calculate Δ*G*_T_*_*_disruption_, we adopted the simplifying assumption that sRNA binding results in a local structural alteration at the site of hybridization, but not longer-range effects on overall target secondary structure. Here, we defined local structural alteration as the breakage of only those target intramolecular base pairs that are required to permit formation of the sRNA:target duplex predicted by the RNAhybrid program. Specifically, Δ*G*_T_*_*_disruption_ was calculated as the energy difference between Δ*G*_T_*_*_before_, the free energy of the original target mRNA structure, and Δ*G*_T_*_*_after_, the free energy of the new locally altered structure (Δ*G*_T_*_*_disruption_ = Δ*G*_T_*_*_before_− Δ*G*_T_*_*_after_). We calculated Δ*G*_T_*_*_before_ by the average energy of the original 1000 structures predicted by Sfold, and Δ*G*_T_*_*_after_ by the average energy of all of the 1000 locally altered structures.

Similarly, Δ*G*_S-disruption_ is the disruption energy for the sRNA, which represents the free energy cost to unpair the RNA's intramolecular base pairs at the site of hybridization. Using 1000 structures predicted by Sfold for the sRNA, Δ*G*_S-disruption_ was computed by the same approach described above for computing Δ*G*_T_*_*_disruption_. Using 1000 structures predicted by Sfold for the sRNA, Δ*G*_hybrid_ is the free energy gain from the hybridization, i.e. the stability of the complete hybrid as computed by the RNAhybrid program. The total energy change of the hybridization is Δ*G*_total_ = Δ*G*_initiation_ + Δ*G*_hybrid_ − (Δ*G*_T-disruption_ + Δ*G*_S-disruption_), where the initiation energy Δ*G*_initiation_ = 4.1 kcal/mol (51), i.e. the energy cost for the initiation of interaction between two RNA molecules. The hybridization was predicted to be successful if the hybridization process was energetically favorable, i.e. Δ*G*_total_ < 0.

The scripts for executing the above computations are available from GitHub at https://github.com/Ding-RNA-Bioinformatics-Lab/Sfold-sRNA. For broad applications, however, we think our prototype method needs further development to incorporate sequence features, evolutionary conservation and possibly heuristic considerations for improving predictive accuracy.

## RESULTS

### Ribo-seq validates most known targets of RyhB

**Figure 1.**
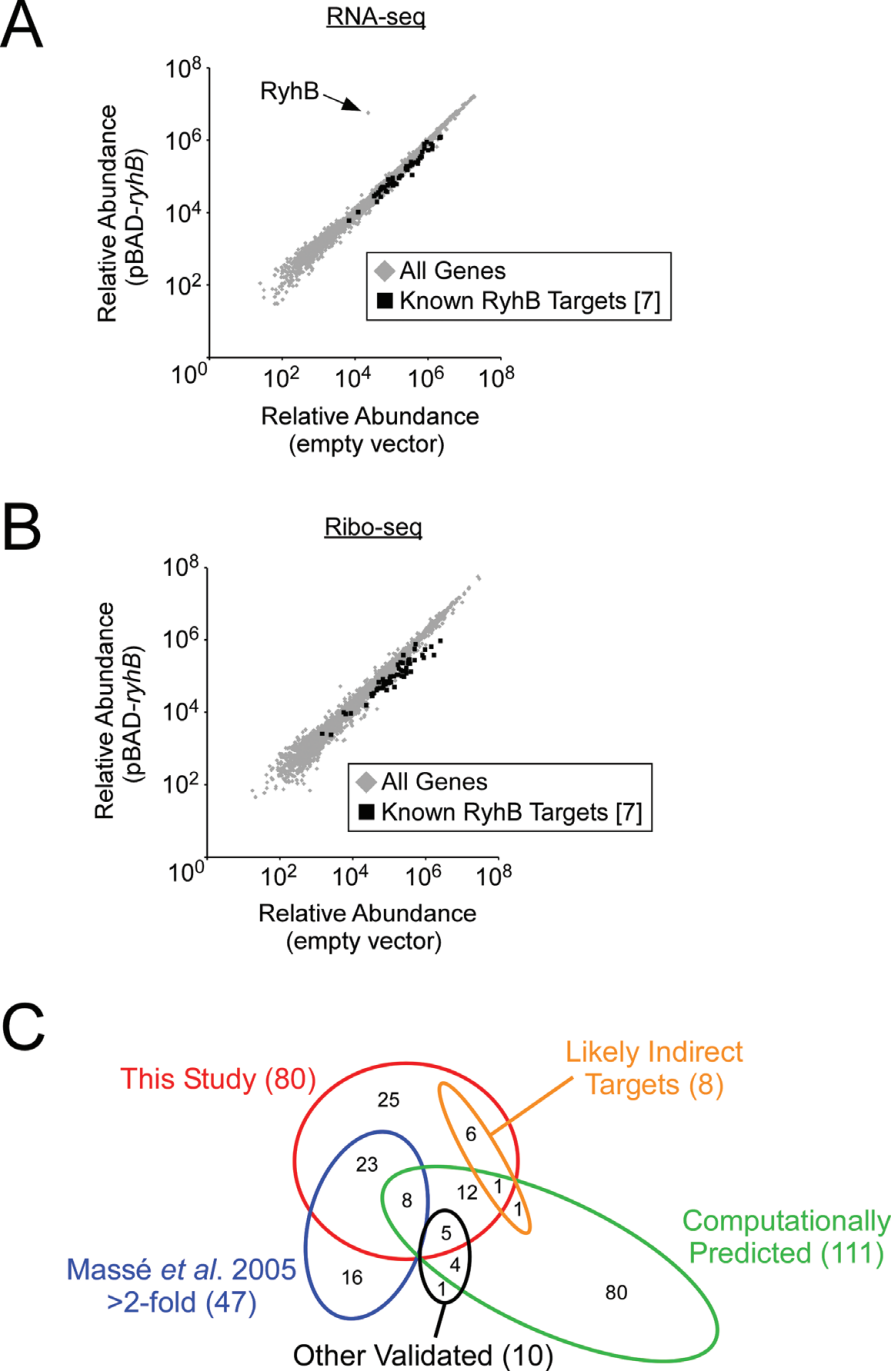
Ribo-seq identifies RyhB targets. **(A)** Scatter-plot showing relative abundance of RNA (from RNA-seq) for all genes in cells expressing RyhB from pBAD-*ryhB* and control cells containing pNM12 (empty vector). Relative abundance of each gene was calculated as the total number of sequence reads mapping to the gene, averaged for two independent biological replicates. Each gene is represented as a gray diamond. Black squares represent genes previously shown to be negatively regulated by RyhB >2-fold by microarray analysis (7). The data-point for RyhB is indicated with an arrow. **(B)** Equivalent scatter-plot showing relative abundance of ribosome-footprinted RNA for all genes. **(C)** Venn diagram showing the overlap among the following classes of genes: putative RyhB target genes identified in this study (‘This Study’); those computationally predicted (‘Computationally Predicted’) (14,19,23); those identified by microarray analysis as direct targets with regulation >2-fold (‘Massé *et al*., 2005 >2-fold’) (7); those identified experimentally using other approaches (‘Other Validated’) (9,10,14,23,59,61), and those identified by microarray analysis as likely indirect targets (‘Likely Indirect Targets’) (7). Numbers in parentheses indicate the number of genes in the corresponding class.

We used Ribo-seq to detect changes in RNA levels and translation levels for all genes in cells transiently expressing RyhB and control cells with empty vector. Transient expression of sRNAs has been shown previously to limit indirect regulatory effects (52), and expression of RyhB for <10 min has been shown to result in regulation of known target mRNAs (7). Regulatory targets of RyhB were identified by comparing total RNA levels (RNA-seq component) or ribosome-footprinted RNA levels (Ribo-seq component) for all genes in RyhB-expressing and control cells (Figure 1). RNA levels and/or translation levels of 80 genes were significantly altered >2-fold by expression of RyhB (Table 1). More targets were identified that were negatively regulated by RyhB (64 genes) than those that were positively regulated (16 genes), consistent with the majority of known RyhB targets being negatively regulated (7). All putative target genes identified using this approach, as well as all other previously described target genes, are listed in Supplementary Table S3.

A previous microarray study using similar experimental conditions identified 47 genes whose RNA level was regulated >2-fold by transient overexpression of RyhB (black squares in Figure 1A, B) (7). We detected >2-fold changes in either the RNA-seq or Ribo-seq data for 31 of these genes. A further 13 genes showed changes of >1.5-fold. 7 of the remaining targets we identified have been previously described as genes that are indirectly regulated by RyhB (7). Thus, our approach identified the majority of described RyhB-regulated genes. Strikingly, most of the previously described targets confirmed by our data showed larger differences in the Ribo-seq data (Figure 1B) than the RNA-seq data (Figure 1A), likely reflecting regulation at the levels of both RNA stability and translation. A summary of the RyhB target genes identified by Ribo-seq and their overlap with targets identified/predicted in other studies is shown in Figure 1C.

### Ribo-seq identifies novel RyhB targets

**Figure 2.**
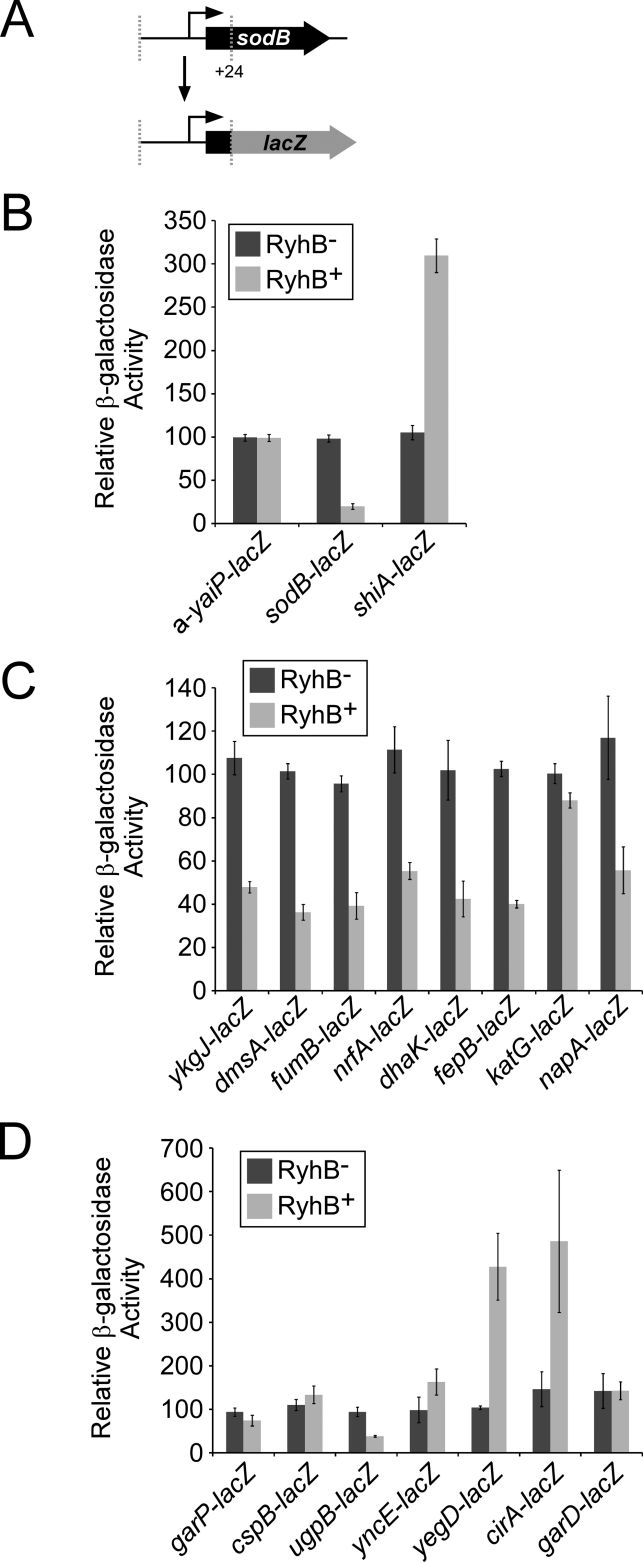
Validation of RyhB targets using *lacZ* reporter gene assays. **(A)** Schematic of *lacZ* translational fusions. Regions upstream of candidate genes, including the first 24 bp of each gene, were fused translationally to *lacZ* in a single-copy plasmid. **(B)** β-galactosidase assays of control *lacZ* fusions in RyhB^−^ (MG1655 Δ*lacZ* Δ*ryhB*; dark gray bars) and RyhB^+^ (MG1655Δ *lacZ*; light gray bars) strains. The antisense RNA opposite *yaiP* is not expected to be RyhB-regulated (i.e. negative control). *sodB* (repressed) and *shiA* (activated) are known RyhB-regulated genes (i.e. positive controls). **(C)** β-galactosidase assays for genes predicted from Ribo-seq data to be repressed by RyhB. **(D)** β-galactosidase assays for genes predicted from Ribo-seq data to be activated by RyhB. β-galactosidase activity was normalized as described in the Materials and Methods.

In addition to previously described RyhB targets, we identified 25 putative novel targets, as well as 13 previously predicted targets (14,19,23). To validate these novel target genes, we constructed translational fusions of the gene and upstream region to the *lacZ* reporter gene for each of 15 selected genes, as well as two positive controls (*sodB* and *shiA*), and one negative control (a-*yaiP*; Figure 2A). The upstream regions included promoters (53) and were typically taken from –300 to +24 bp relative to the translation start. In a few cases, the upstream boundary was extended based on promoter predictions from the RNA-seq data. We then measured β-galactosidase activity from these plasmids in MG1655 Δ*lacZ* (RyhB^+^) and a *ryhB* gene deletion derivative of MG1655 Δ*lacZ* (RyhB^−^). Since in *E. coli*, RyhB is expressed in iron-limiting conditions (6), β-galactosidase assays were performed in M9 minimal medium. As expected, we observed repression of the *sodB-lacZ* translation fusion and activation of *shiA-lacZ* translation fusion by RyhB (Figure 2B), as has been described previously (6,10). A transcriptional *lacZ* fusion of an antisense RNA within the *yaiP* gene (39) represents a negative control (not known to be regulated by RyhB and not identified by Ribo-seq); as expected, we observed no regulation by RyhB (Figure 2B). We next analyzed expression of translational *lacZ* fusions for eight genes predicted from the Ribo-seq data to be negatively regulated by RyhB. All but one of these fusions (*katG-lacZ*) were expressed at a substantially lower level in the RyhB^+^ strain than in the RyhB^−^ strain (Figure 2C). *katG-lacZ* expression was significantly lower in the RyhB^+^ strain than in the RyhB^−^ strain, but the difference in expression was modest.

We next analyzed expression of translational *lacZ* fusions for seven genes predicted from the Ribo-seq data to be positively regulated by RyhB. Three of these fusions (*cirA-lacZ*, *yncE-lacZ*, and *yegD-lacZ*) were expressed at a significantly higher level in the RyhB^+^ strain than in the RyhB^−^ strain (Figure 2D). Among these genes, *cirA* has been recently reported as a positively regulated target of RyhB (Salvail et al. 2013). Surprisingly, one of the fusions for a putative RyhB-activated gene, *ugpB*, showed significantly lower expression in the RyhB^+^ strain than in the RyhB^−^ strain (Figure 2D), suggesting that RyhB can have opposite regulatory effects depending on the growth conditions. We also analyzed expression of translational *lacZ* fusions for five genes predicted from the Ribo-seq data to be positively regulated by RyhB, but for which the fold difference between RyhB^+^ and RyhB^−^ cells was <2-fold (Supplementary Figure S1). Only one of these fusions, *eptB-lacZ*, showed significantly higher expression in the RyhB^+^ strain than in the RyhB^−^ strain (Supplementary Figure S1B), suggesting there are few false negatives in the set of RyhB-activated genes identified by Ribo-seq using a cut-off of two-fold.

The *lacZ* fusion constructs described above include the corresponding promoter for the putative RyhB-regulated gene. Hence, it is possible that effects of RyhB on expression of the *lacZ* fusions could be due to altered levels of transcription initiation. To rule out this possibility, we selected eight of the RyhB-regulated genes and constructed reporter plasmids in which a constitutive promoter (54) was fused to the 5′ UTR and first 24 nt of the ORF for each gene, which was in turn fused translationally to *lacZ* (Supplementary Figure S2A). All of these fusions were expressed at a substantially lower or higher level in the RyhB^+^ strain than in the RyhB^−^ strain (Supplementary Figure S2B), consistent with the activity of longer reporter fusions (Figure 2). Thus, these data indicate post-transcriptional regulation by RyhB. We also analyzed expression of translational *lacZ* fusions of *ynfF* and *fumB*, putative RyhB-repressed genes that are predicted to be located within multi-gene operons (55,56), consistent with our RNA-seq data. Hence, we fused a constitutive promoter (54) to the region surrounding the start of these genes, which in turn was fused to *lacZ* (Supplementary Figure S2C). As for all other putative RyhB-repressed genes tested, expression of *fumB-lacZ* and *ynfF-lacZ* was substantially lower in the RyhB^+^ strain than in the RyhB^−^ strain (Supplementary Figure S2D).

### RyhB regulation of katG requires sequence within the ORF

**Figure 3.**
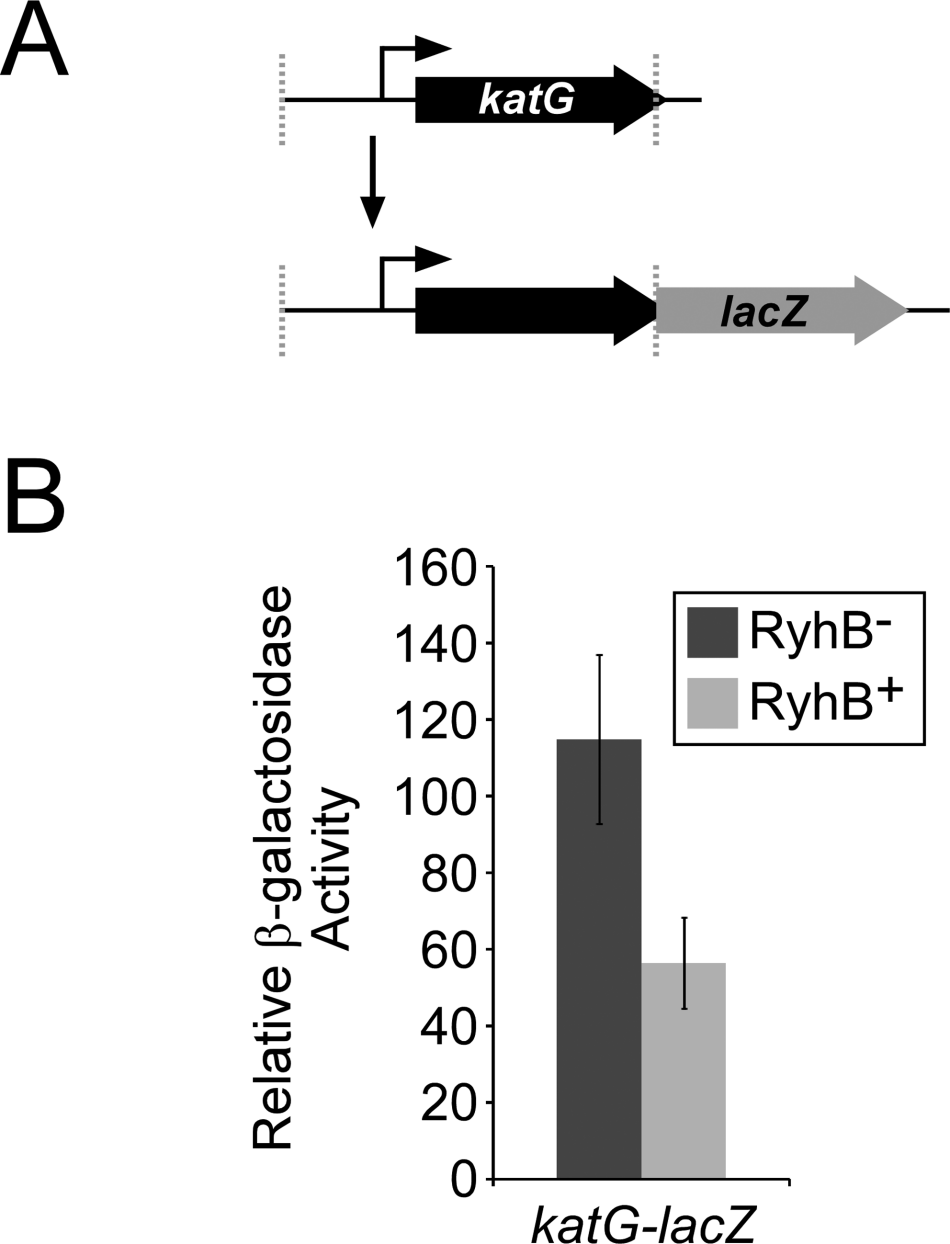
Repression of *katG* by RyhB requires sequence in the ORF. (**A**) Schematic of the long translational fusion of *katG* to *lacZ*. The region from 350 upstream of *katG* to the base immediately before the stop codon of *katG* was fused translationally to *lacZ* in a single-copy plasmid. (**B**) β-galactosidase assays of the long translational fusion of *katG* in RyhB^−^ (MG1655 Δ*lacZ* Δ*ryhB*; dark gray bars) and RyhB^+^ (MG1655Δ *lacZ*; light gray bars) strains. β-galactosidase activity was normalized as described in the Materials and Methods.

Although we detected slight repression of *katG-lacZ* by RyhB (Figure 2C), this was substantially less than the change (2.3-fold) observed in the Ribo-seq data set (Table 2), and is also substantially less than for any other tested gene (Figure 2C). Moreover, KatG binds iron in the form of heme (57). Hence, we hypothesized that *katG* is a genuine target of RyhB but that base-pairing of RyhB with sequences within the coding sequence of *katG* is required for full repression. To test this hypothesis, the upstream region and entire *katG* gene were translationally fused to *lacZ* (Figure 3A). In the context of this fusion, the level of *katG* repression was substantially higher than that with the shorter *lacZ* fusion that contains only 25 bp of the coding region (Figures 2C and 3B), consistent with the data from Ribo-seq.

**Table 2.** Hybridization energies (in kcal/mol) for predicted RyhB binding sites in the *fepB* 5′ UTR

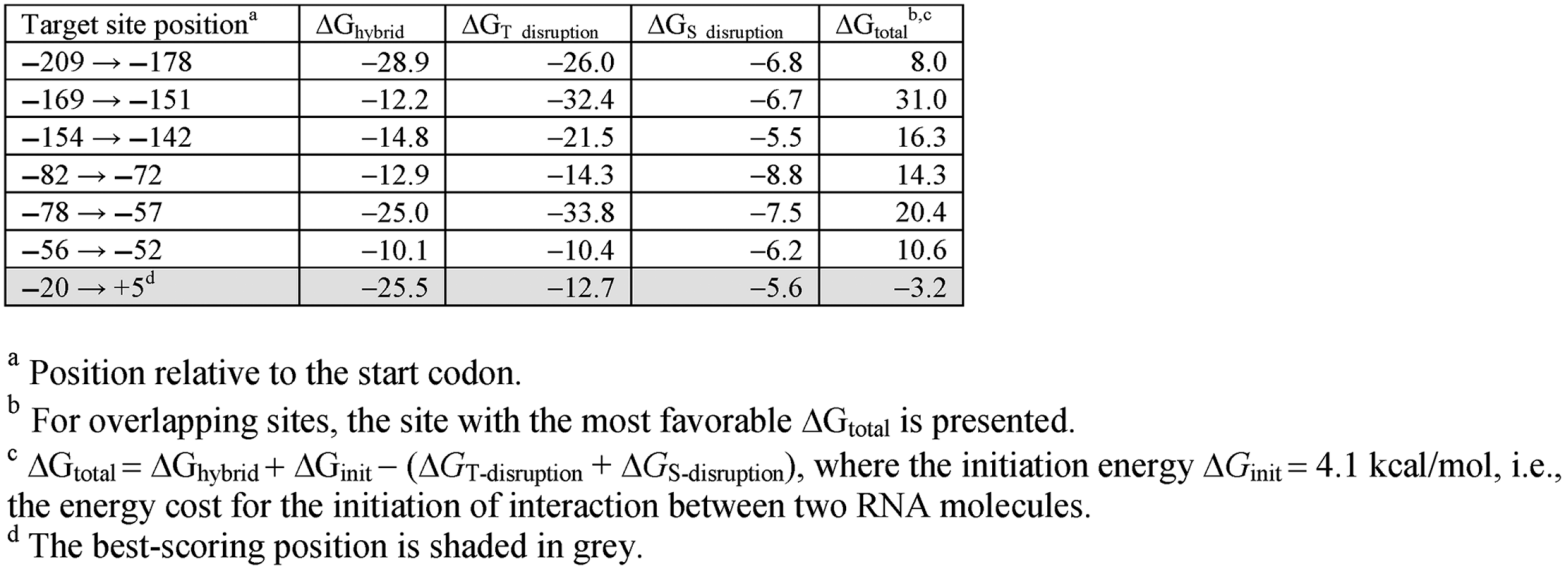				

### Ribo-seq correctly identifies translational targets of RyhB

**Figure 4.**
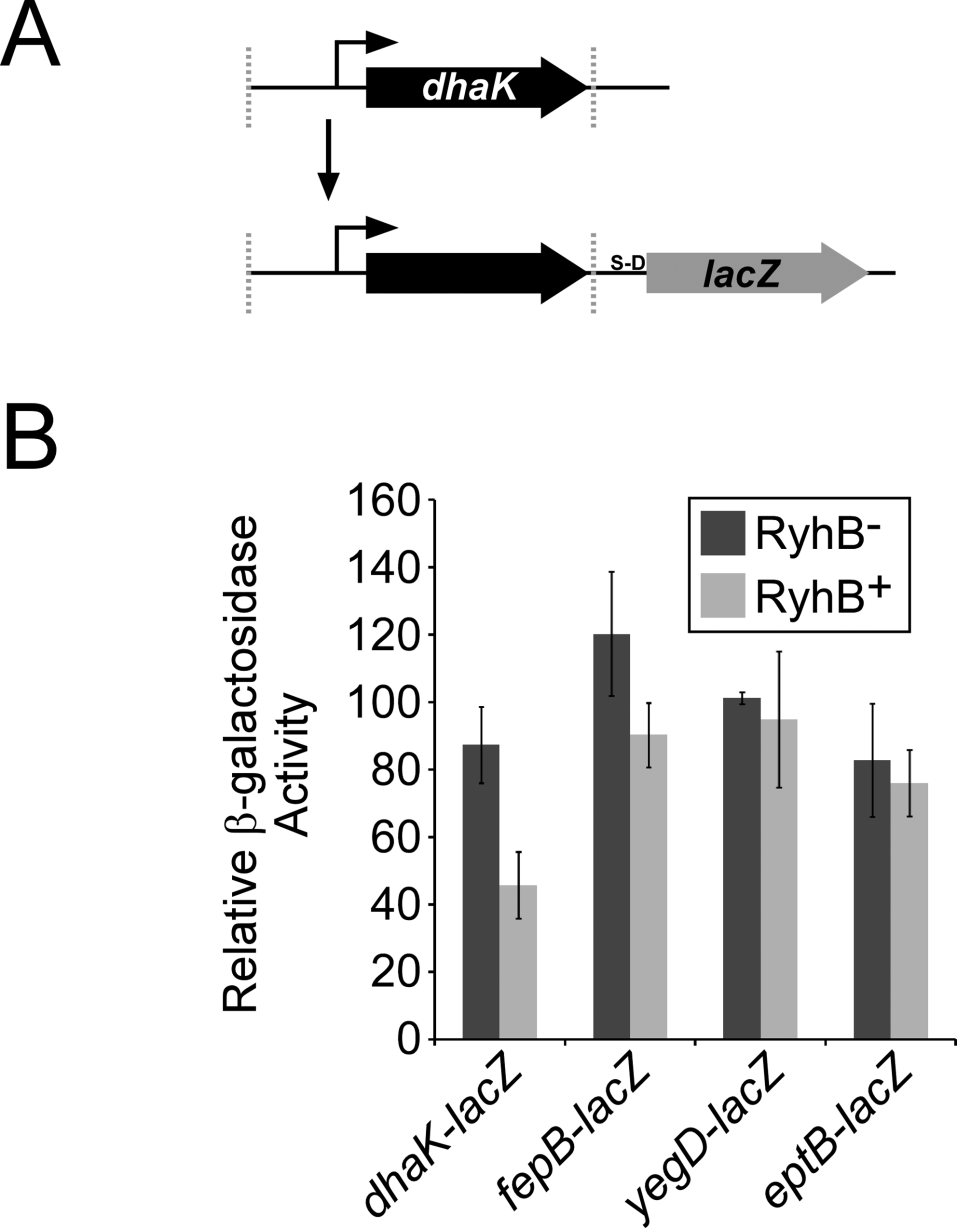
Ribo-seq accurately distinguishes regulation of RNA stability from regulation of translation. (**A**) Schematic of transcriptional fusions to *lacZ*. Regions beginning upstream of genes and including the entire gene were fused transcriptionally to *lacZ* (i.e. *lacZ* has its own Shine-Dalgarno sequence) in a single-copy plasmid. (**B**) β-galactosidase assays of *dhaK, fepB, yegD*, and *eptB* transcriptional fusions in RyhB^−^ (MG1655 Δ*lacZ* Δ*ryhB*; dark gray bars) and RyhB^+^ (MG1655Δ *lacZ*; light gray bars) strains. β-galactosidase activity was normalized as described in the Materials and Methods.

Transcriptomic analysis using microarrays or RNA-seq is a common approach for identification of sRNA targets. However, transcriptomic analysis is unable to identify regulatory effects that occur solely at the level of translation. As indicated in Table 2, some putative RyhB targets identified by Ribo-seq showed a substantially larger change in ribosome-footprinted RNA than in total RNA levels. We hypothesized that such targets are regulated by RyhB predominantly at the translational level. To test this hypothesis, we constructed transcriptional fusions to *lacZ* for four of the validated targets (Figure 4A). Three of these genes (*fepB*, *yegD* and *eptB*) were predicted from Ribo-seq data to be regulated predominantly at the level of translation. The fourth (*dhaK*) was predicted to be regulated predominantly at the level of mRNA stability. We compared expression of *lacZ* for each fusion in RyhB^+^ and RyhB^−^ strains. Consistent with the Ribo-seq data, expression of the *dhaK* transcriptional *lacZ* fusion was significantly repressed by RyhB, whereas repression of the *fepB* fusion by RyhB was significantly lower than for the translational fusion (cf. Figures 2C and 4B). Moreover, no activation of the *yegD* and *eptB* transcriptional fusions by RyhB was observed, despite significant activation of the equivalent translational fusions (cf. Figures 2D and 4B, and Supplementary Figure S2B). We conclude that Ribo-seq can be used to reliably predict the mode (i.e. RNA stability or translation) of regulation by sRNAs.

### Confirmation that fepB is a direct target of RyhB

**Figure 5.**
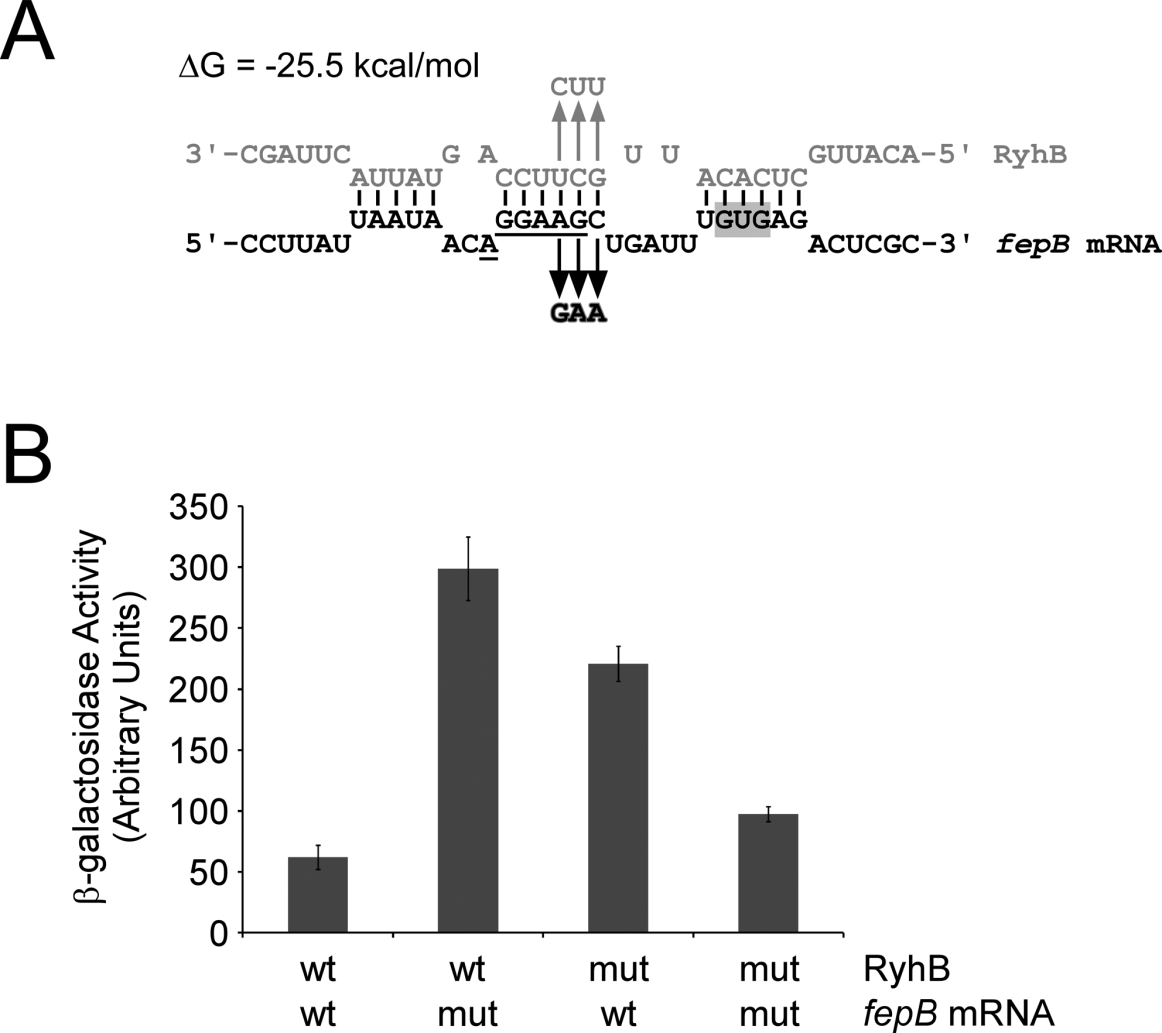
RyhB directly represses translation of *fepB* by base-pairing with the ribosome binding site. (**A**) Prediction of base-pairing interaction between RyhB and *fepB* mRNA. For the predicted site of the interaction, the starting and ending nucleotides are U^−20^ and G^5^ for *fepB* mRNA (nucleotide positions relative to start codon), and C^45^ and A^65^ for RyhB. The translation start codon of *fepB* is shaded in gray. The putative Shine-Dalgarno sequence is underlined. The arrows indicate the changes in the mutant RNAs. (**B**) β-galactosidase assays of wild-type (wt) and mutant (mut) *fepB* translational fusions to *lacZ* in cells expressing wild-type (wt) or mutant (mut) RyhB, as indicated. β-galactosidase activity was calculated as described previously (38).

RyhB is known to positively regulate two genes, *shiA* or *cirA*, at the level of translation (9,10). Two genes, *acnB* and *cysE*, have been described as being translationally repressed by RyhB (58,59). Our Ribo-seq and *lacZ* fusion data indicated that *fepB* is also translationally repressed by RyhB (Table 1; Figures 2C and 4B), although we cannot rule that the possibility that the regulation could be due to indirect effects. To investigate this further, we developed a new computational approach (Materials and Methods) to identify potential base-pairing sites of RyhB within *fepB*. We used this method to identify a potential base-pairing site of RyhB overlapping the S-D sequence of *fepB* (Figure 5A and the highlighted row in Table 2). This is the only site within the *fepB* 5′ UTR that is predicted to have a favorable total energy change for the hybridization (i.e. Δ*G*_total_ < 0 kcal/mol; see Materials and Methods). To determine whether this is a genuine site of RyhB base-pairing, we introduced a mutation in the *fepB* mRNA that is predicted to disrupt *fepB*-RyhB pairing (Figure 5A), in the context of the translational *lacZ* fusion construct (Figure 2A). We also constructed a strain that expresses a mutant RyhB that is predicted to base-pair with this mutant *fepB* mRNA, but not with the wild-type transcript (Figure 5A). We measured β-galactosidase activity in strains with the wild-type RyhB and the wild-type *lacZ* fusion, and in strains with each of the mutations (in RyhB or *fepB* mRNA) alone or in combination (Figure 5B). β-galactosidase activity was higher with mutant RyhB or mutant *fepB-lacZ*, relative to the wild-type RNAs (note that changes to the S-D sequence may improve its affinity for the 30S ribosome, accounting for the increased expression of the mutant *fepB* mRNA). However, combining mutant RyhB and mutant *fepB-lacZ* resulted in β-galactosidase activity similar to that of fully wild-type cells (Figure 5B). We conclude that RyhB directly represses translation of *fepB* by base-pairing with the region around the Shine-Dalgarno sequence. We similarly confirmed direct activation of *cirA* translation by RyhB (Supplementary Figure S3), consistent with an earlier report (9).

## DISCUSSION

### Ribo-seq is sensitive and specific

Ribo-seq is a state-of-the-art technology that enables comprehensive and quantitative measurements of translation (25,60). Our study represents the first detailed assessment of Ribo-seq as a method for identifying regulatory targets of sRNAs. We selected RyhB due to the wealth of information on this sRNA, including a large number of previously identified and predicted targets (6,7,9,10,14,19,59,61). These existing data allowed us to accurately assess the number of true/false positives and true/false negatives in our own data set (Figure 1C). 25 of the RyhB target genes we identified have not been identified or predicted previously. We tested 11 of these using reporter gene fusions (Figures 2 and 3) and confirmed regulation for seven. Extrapolating from this number, we estimate that only ≈9 of the 80 RyhB-regulated genes we identified are false positives. We also compared our list of RyhB-regulated genes to only those genes that have been experimentally confirmed in other studies (6,7,9,10,14,23,59,61). We identified 36/57 of these genes, suggesting that our data have at least 21 false negatives. Consistent with there being false negatives in our data, 13 of the 47 genes identified in a previous microarray study (7) showed changes in RNA and/or translation levels of between 1.5-fold and 2-fold in our data. Nonetheless, it is reasonable to assume that other published data sets include false positives. Therefore, we believe the sensitivity of our approach to be >63% (36 identified positives/57 true positives).

It is unclear if the source of false positives in our data is technical or if it reflects a biological phenomenon. Previous studies of RyhB target genes also identified several false positive genes that are likely to be indirectly regulated as a consequence of changes in free iron levels following RyhB expression, leading to altered activity of the Fur transcription factor (7,62). We identified seven of the same genes (Table 1; orange set in Figure 1C), as well as novel target genes, *fepB* and *yncE*, whose transcription is known to be Fur-regulated (63). However, it is important to note that our data clearly indicate that *fepB* is a direct target of RyhB (Figure 5). In both our study and (7), RyhB expression was limited to a short period of time (10 min in our study and 15 min in (7)). Transient expression of the sRNA for a short period of time should limit the potential for indirect effects (24,52), but may not be sufficient to avoid them completely. The growth media used in our study may also be a source of false positives. For the Ribo-seq experiment, cells were grown in rich medium (LB), but for validation experiments with reporter gene fusions, cells were grown in minimal medium (M9). It is possible that some genes that appear to be false positives are regulated by RyhB in a condition-specific manner. Consistent with this hypothesis, a recent study showed that *acnB* is regulated by RyhB only when iron levels are high (58).

We identified a substantially higher ratio of false positives for genes that were predicted by Ribo-seq to be activated by RyhB. We believe this bias simply reflects the fact that most RyhB-regulated genes are repressed (Table 1). In contrast, false positives are expected to be distributed evenly between putative activated and repressed genes. Hence, a higher proportion of false positives is expected for genes that are predicted by Ribo-seq to be RyhB-activated.

### Ribo-seq identifies translationally-regulated genes

The major theoretical advantage of Ribo-seq over other transcription profiling is the ability to identify regulation at the level of translation. We identified many putative translational targets of RyhB, including examples of both positive and negative regulation (Figure 2). We successfully validated three out of three tested examples (Figure 4). We conclude that Ribo-seq is an effective method for detecting regulation by sRNAs at all levels (i.e. transcription, mRNA stability, translation).

### Novel RyhB targets suggest additional roles and new mechanisms of regulation

The primary function of RyhB is in the iron-sparing response: RyhB represses expression of non-essential genes that encode iron-associated proteins. Of the 27 novel RyhB-repressed genes we identified using Ribo-seq, 13 are known to encode iron-bound proteins. In particular, genes encoding every subunit of the iron-sulfur cluster-containing NapABCGH periplasmic nitrate reductase were observed to be down-regulated by RyhB, largely at the level of mRNA stability (Table 1). Four RyhB-repressed genes, *dmsA*, *ynfF*, *nagT*, and *fumB*, also encode proteins that contain iron-sulfur clusters or are part of a complex that contains iron-sulfur clusters (64–67). Two other RyhB-repressed genes, *nrfA* and *katG*, encode heme-binding proteins (57,68). Thus, the known function of RyhB in iron sparing extends to repression of many novel target genes. Other putative RyhB-repressed genes identified by Ribo-seq have either no known function, or have not been previously associated with iron utilization. These include *dhaK* and *dhaM*, which both encode subunits of dihydroxyacetone kinase.

Only one RyhB-activated gene, *yncE*, has a well-established association with iron utilization, although *cpdA* function has been tentatively linked to iron (69). *yncE* is repressed by Fur, the iron-sensing transcription factor (63), and has been predicted to be a component of an iron transporter (63,70). Thus, RyhB may up-regulate expression of an iron transporter in response to conditions of iron starvation. In contrast, *fepB*, which encodes a ferric enterobactin transporter, is repressed by both Fur and RyhB. Hence, the impact of iron starvation on iron uptake by ferric enterobactin is unclear, since *fepB* would be simultaneously relieved of Fur repression and repressed by RyhB.

In addition to identifying physiological functions of RyhB, our data also provide insight into mechanisms of RyhB-mediated regulation. For example, our data indicate that translational repression of *fepB* by RyhB is due to base-pairing around the S-D sequence and start codon, similar to regulation of other RyhB target genes (59,71). In contrast, our data suggest that RyhB regulation of *katG* occurs through a novel regulatory mechanism. *katG* is repressed by RyhB (Figure 2), but maximal repression requires sequence within the *katG* ORF, downstream of the eighth codon (Figure 3). This suggests that RyhB base-pairs, at least in part, with the *katG* coding sequence. There are described examples of sRNAs that regulate by base-pairing with mRNA coding sequence, far from the translational start (16,72,73). However, in every case tested, regulation is at the level of mRNA stability, whereas *katG* is translationally repressed by RyhB (Table 2). We propose that RyhB represses translation of *katG* through a novel mechanism that involves base-pairing with the *katG* coding sequence and alteration of long-range intramolecular base-pairing in target RNA secondary structure.

### Ribo-seq data can facilitate development of improved sRNA target prediction tools

sRNA target recognition is known to be associated with low sRNA and mRNA secondary structure (12,13), the presence of Hfq binding sites in the mRNA (12), the preference for a sRNA 5′ domain of at least seven bp for Watson-Crick base-pairing with target sequence (15,16), and sequence conservation of the sRNA and mRNA (13,14). These features can be useful for target prediction, and several programs have been developed that incorporate some of these features (14,19–23). Existing programs can correctly identify many genuine sRNA target genes (14,19–23); however, they have high false negative and false positive rates. We analyzed our Ribo-seq data for previous computational predictions of RyhB target genes (Figure 1C; Supplementary Table S3) (14,19,23). The majority of predicted targets lack a detectable level of regulation (Figure 1C), indicating high false positive rate for the predictions. The majority of targets identified by Ribo-seq have not been previously predicted, indicating a high number of false negatives (14,19,23). The Ribo-seq data sets will be a valuable resource for improvement of sRNA target prediction, just as many genome-scale data sets for metazoan microRNAs have significantly improved their target predictions (74).

Importantly, use of data from comprehensive experimental approaches such as Ribo-seq still requires downstream computational analysis and prediction. Specifically, Ribo-Seq can identify the target mRNAs but not the precise site of sRNA:target hybridization. Hence, sRNA target prediction algorithms could be combined with Ribo-seq data sets. This will greatly facilitate target site prediction, since the predictions will be focused on one mRNA rather than the whole transcriptome.

## CONCLUSIONS

Our work highlights the value of Ribo-seq for identifying sRNA targets, especially for genes regulated at the level of translation. Ribo-seq can be easily adaptable to the studies of other sRNAs, including those in other bacteria species, making comprehensive, genome-scale investigation of sRNA targets experimentally accessible. Ribo-seq analysis of sRNA targets promises to be valuable for refining the ‘rules’ of sRNA target recognition.

## Supplementary Material

SUPPLEMENTARY DATA
