# Supplementary material for: Identification of bacterial sRNA regulatory targets using ribosome profiling
Source: Nucleic Acids Res. 2015 Nov 5;43(21):10308–20. doi: 10.1093/nar/gkv1158 (PMC4666370; doi:10.1093/nar/gkv1158)
Supplement: SUPPLEMENTARY DATA [file supp_43_21_10308__index.html]

Identification of bacterial sRNA regulatory targets using ribosome profiling — SUPPLEMENTARY DATA 

# Identification of bacterial sRNA regulatory targets using ribosome profiling

## SUPPLEMENTARY DATA

- SUPPLEMENTARY DATA
- SUPPLEMENTARY DATA
- SUPPLEMENTARY DATA
- SUPPLEMENTARY DATA
