# Supplementary material for: Identification of bacterial sRNA regulatory targets using ribosome profiling
Source: Nucleic Acids Res. 2015 Nov 5;43(21):10308–20. doi: 10.1093/nar/gkv1158 (PMC4666370; doi:10.1093/nar/gkv1158)
Supplement: SUPPLEMENTARY DATA [file supp_gkv1158_nar-03489-a-2014-File008.pdf]

SUPPLEMENTARY FIGURE LEGENDS

Figure S1. Validation of RyhB repression of *ynfF*. (A) Schematic of *lacZ* translational fusion for *ynfF*, a gene predicted by Ribo-seq to be repressed by RyhB. A constitutive promoter from pAMD001 was fused to the region around the start of the *ynfF* gene and this was fused to *lacZ* in a single-copy plasmid. **(B)** β-galactosidase assays of the *ynfF lacZ* fusion. Data are shown for $RyhB^-$ (MG1655 Δ*lacZ* Δ*ryhB*; dark gray bars) and $RyhB^+$ (MG1655Δ *lacZ*; light gray bars) strains. β-galactosidase activity was normalized as described in the Materials and Methods.

Figure S2. Validation of genes predicted by Ribo-seq to be weakly activated by RyhB. (A) Schematic of *lacZ* translational fusions. Regions upstream of candidate genes, including the first 24 bp of the gene, were fused translationally to *lacZ* in a single-copy plasmid. **(B)** β-galactosidase assays of *lacZ* fusions for genes predicted from Ribo-seq data to be weakly repressed by RyhB. Data are shown for $RyhB^-$ (MG1655 Δ*lacZ* Δ*ryhB*; dark gray bars) and $RyhB^+$ (MG1655Δ *lacZ*; light gray bars) strains. β-galactosidase activity was normalized as described in the Materials and Methods.

Figure S3. RyhB directly activates translation of *cirA* by base-pairing with the 5’ UTR. (A) Prediction of base-pairing interactions between RyhB and the *cirA* 5’ UTR. The arrows indicate the changes in the mutant RNAs. (B) β-galactosidase assays of wild-type and mutant *cirA* translational fusions to *lacZ* in cells expressing wild-type or mutant RyhB, as indicated. β-galactosidase activity was calculated as described previously (60).
