# Supplementary figures and images for: Identification of bacterial sRNA regulatory targets using ribosome profiling
Source: Nucleic Acids Res. 2015 Nov 5;43(21):10308–20. doi: 10.1093/nar/gkv1158 (PMC4666370; doi:10.1093/nar/gkv1158)

A

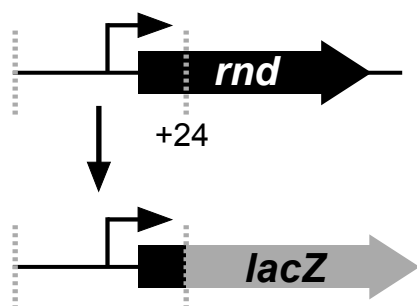

B

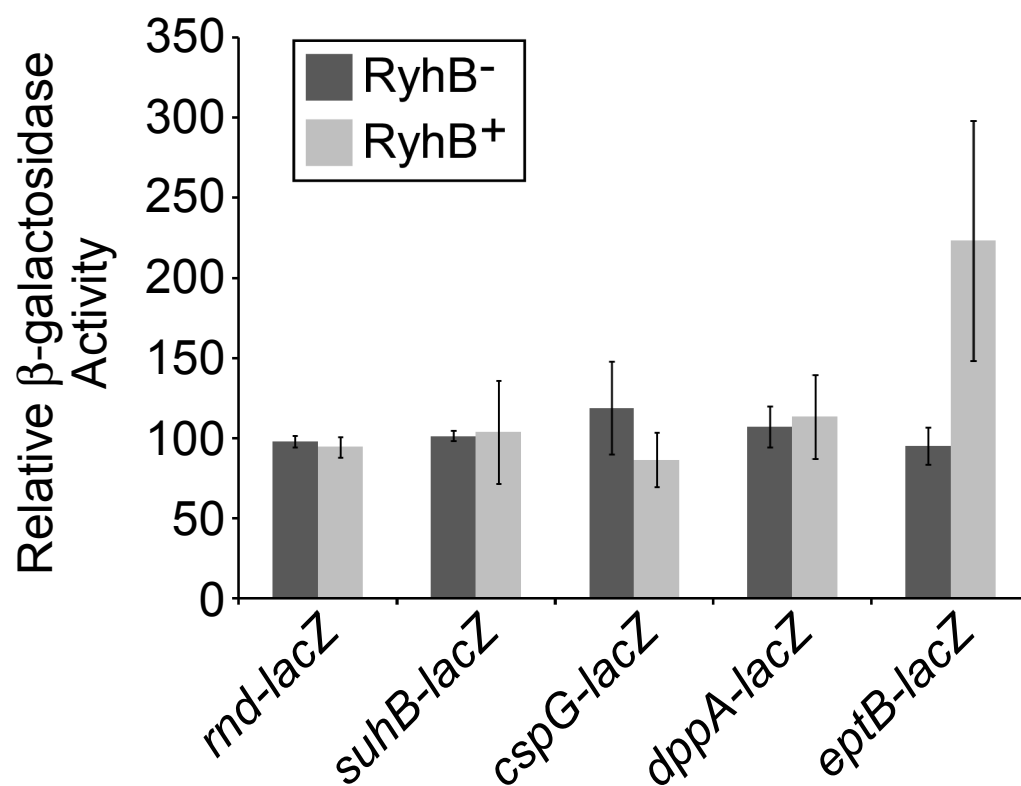

A

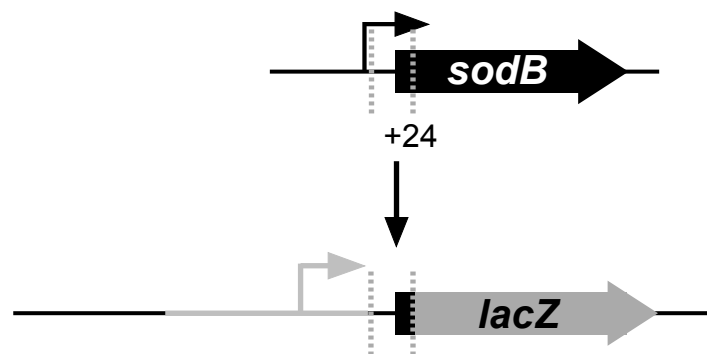

B

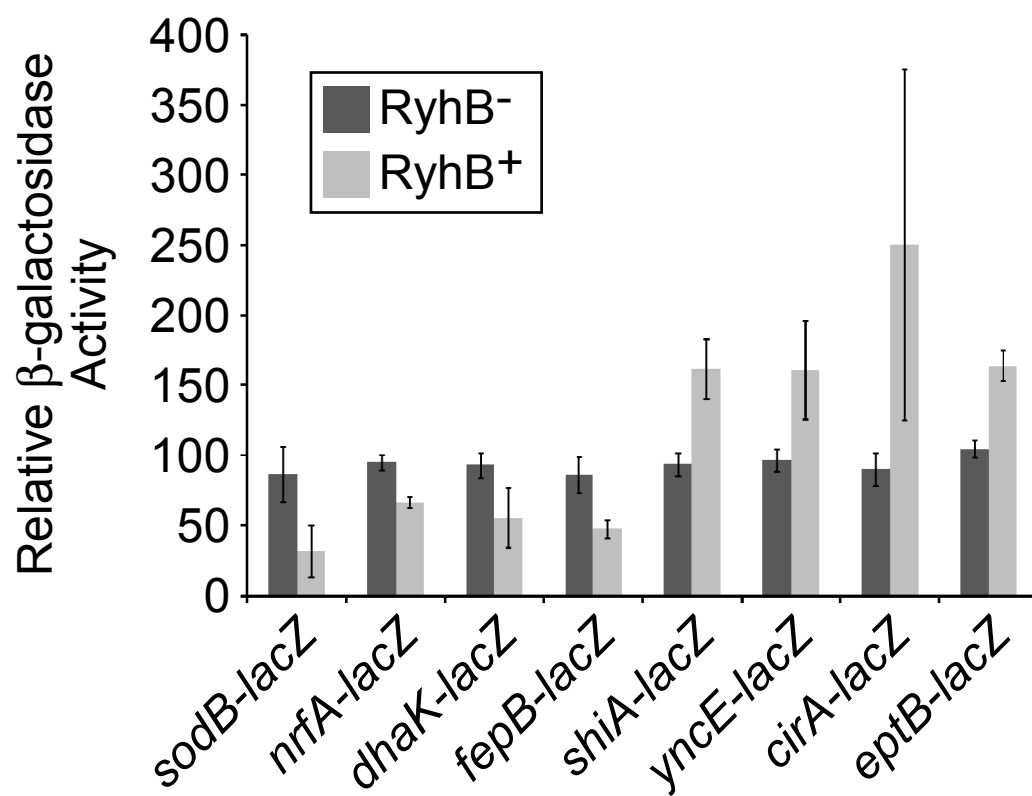

C

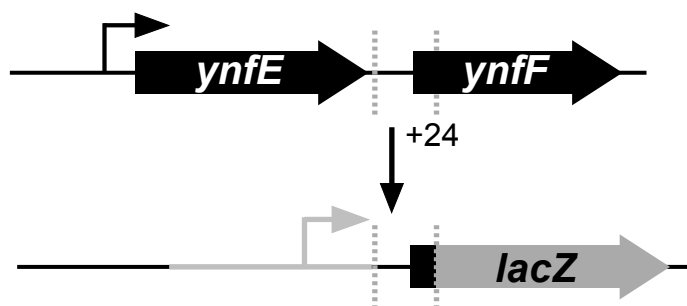

D

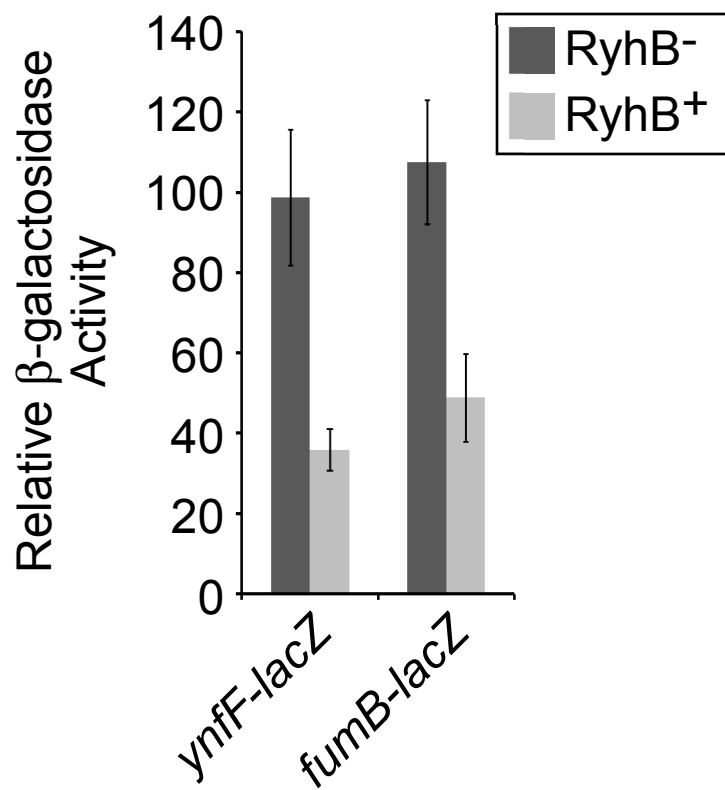

A

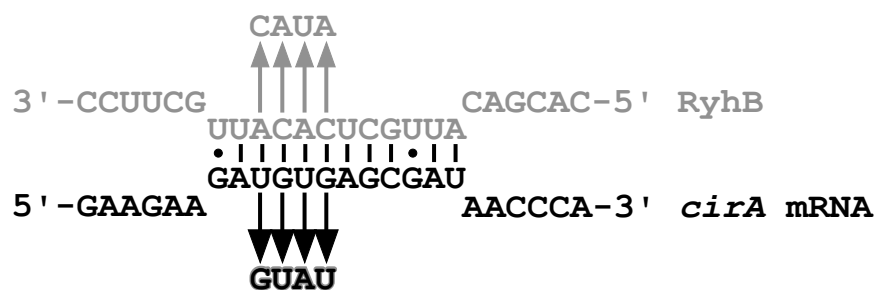

B

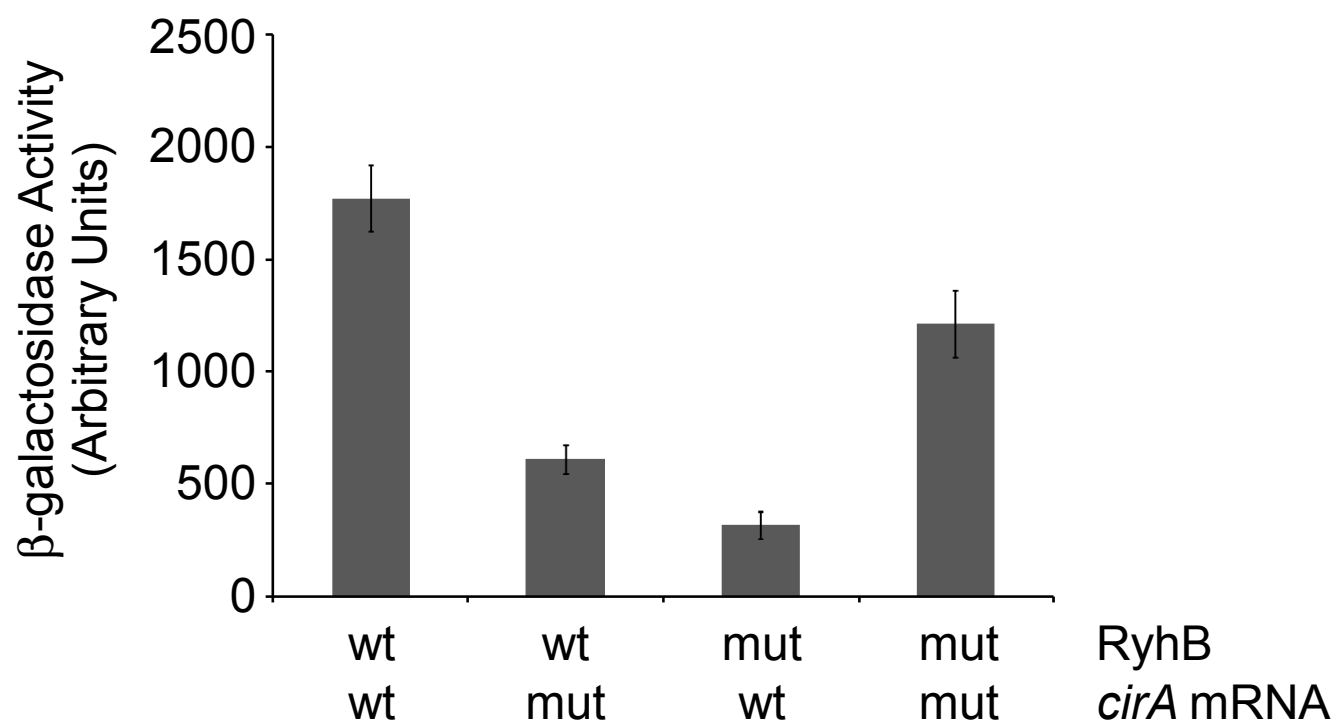

Supplement: SUPPLEMENTARY DATA [file supp_gkv1158_nar-03489-a-2014-File009.pdf]
