# Supplementary material for: Identification of bacterial sRNA regulatory targets using ribosome profiling
Source: Nucleic Acids Res. 2015 Nov 5;43(21):10308–20. doi: 10.1093/nar/gkv1158 (PMC4666370; doi:10.1093/nar/gkv1158)
Supplement: SUPPLEMENTARY DATA [file supp_gkv1158_nar-03489-a-2014-File010.pdf]

Table S1. Bacterial strains and plasmids used in this study

Strain/Plasmid	Description	Source/Reference
***E.coli* Strains**		
MG1655	F^{-} λ^{-} Δ*ilvG rfb-50 rph-1*	(55)
KP1174	*rne*131*zce*-726::Tn10 Hfq-3×FLAG *araB*::*kan*^{R} *ryhB*::*cat*	This study
MPC144	KP1174 + pNM12	This study
KP1184	KP1174 + pBAD-*ryhB*	This study
AMD052	MG1655 Δ*thyA*	(33)
AMD054	MG1655 Δ*lacZ*	(38)
JXW026	MG1655 Δ*lacZ* Δ*ryhB*	This study
JXW028	MG1655 Δ*lacZ* with *ryhB* mutation antisense to *cirA* mutation	This study
JXW029	MG1655 Δ*lacZ* with *ryhB* mutation antisense to *fepB* mutation	This study
**Plasmids**		
pNM12	The *ryhB*$^{-}$ control vector; ampicillin resistance	(7)
pBAD-*ryhB*	*ryhB*$^{+}$; ampicillin resistance	(7)
pAMD-BA-*lacZ*	Single-copy *lacZ* fusion vector, chloramphenicol resistance	(38)
pAMD001	*thyA* gene cloned into pGEM-T vector (Promega), ampicillin resistance	(33)
pKD46	Encodes λ recombinase system	(36)
Translational fusions to *lacZ*		
pJXW009	pAMD-BA-*lacZ* with *sodB* upstream sequence from −300 to +24 (relative to translation start site)	This study
pJXW010	pAMD-BA-lacZ with *ykgJ* upstream sequence from −300 to +24 (relative to translation start site)	This study
pJXW011	pAMD-BA-*lacZ* with *dmsA* upstream sequence from −300 to +24 (relative to translation start site)	This study
pJXW013	pAMD-BA-*lacZ* with *nrfA* upstream sequence from −300 to +24 (relative to translation start site)	This study
pJXW014	pAMD-BA-*lacZ* with *dhaK* upstream sequence from −300 to +24 (relative to translation start site)	This study
pJXW015	pAMD-BA-*lacZ* with *fepB* upstream sequence from −300 to +24 (relative to translation start site)	This study
pJXW016	pAMD-BA-*lacZ* with *katG* upstream sequence from −350 to +24 (relative to translation start site)	This study
pJXW017	pAMD-BA-*lacZ* with *napA* upstream sequence from −900 to +24 (relative to translation start site)	This study
pJXW018	pAMD-BA-*lacZ* with *shiA* upstream sequence from −300 to +24 (relative to translation start site)	This study

pJXW019	pAMD-BA-*lacZ* with *garP* upstream sequence from −400 to +24 (relative to translation start site)	This study
pJXW020	pAMD-BA-*lacZ* with *cspB* upstream sequence from −300 to +24 (relative to translation start site)	This study
pJXW021	pAMD-BA-*lacZ* with *ugpB* upstream sequence from −300 to +24 (relative to translation start site)	This study
pJXW022	pAMD-BA-*lacZ* with *yncE* upstream sequence from −300 to +24 (relative to translation start site)	This study
pJXW023	pAMD-BA-*lacZ* with *yegD* upstream sequence from −300 to +24 (relative to translation start site)	This study
pJXW024	pAMD-BA-*lacZ* with *cirA* upstream sequence from −300 to +24 (relative to translation start site)	This study
pJXW025	pAMD-BA-*lacZ* with *suhB* upstream sequence from −300 to +24 (relative to translation start site)	This study
pJXW026	pAMD-BA-*lacZ* with *cspG* upstream sequence from −300 to +24 (relative to translation start site)	This study
pJXW027	pAMD-BA-*lacZ* with *dppA* upstream sequence from −300 to +24 (relative to translation start site)	This study
pJXW028	pAMD-BA-*lacZ* with *eptB* upstream sequence from −300 to +24 (relative to translation start site)	This study
pJXW029	pAMD-BA-*lacZ* with *rnd* upstream sequence from −650 to +24 (relative to translation start site)	This study
pJXW030	pAMD-BA-*lacZ* with *garD* upstream sequence from −300 to +24 (relative to translation start site)	This study
pJXW031	pAMD-BA-*lacZ* with *ynfF* upstream sequence from −60 to +24 (relative to translation start site) and constitutive promoter from pAMD001	This study
pJXW032	pAMD-BA-*lacZ* with *katG* sequence from −350 (relative to translation start site) to the codon immediately before the stop codon	This study
pJXW033	pAMD-BA-*lacZ* with *fumB* upstream sequence from −77 to +24 (relative to translation start site) and constitutive promoter from pAMD001	This study
pJXW034	pAMD-BA-*lacZ* with *nrfA* upstream sequence from −93 to +24 (relative to translation start site) and constitutive promoter from pAMD001	This study
pJXW035	pAMD-BA-*lacZ* with *dhaK* upstream sequence from −106 to +24 (relative to translation start site) and constitutive promoter from pAMD001	This study
pJXW036	pAMD-BA-*lacZ* with *fepB* upstream sequence from −217 to +24 (relative to translation start site) and constitutive promoter from pAMD001	This study
pJXW037	pAMD-BA-*lacZ* with *katG* upstream sequence from −23 (relative to translation start site) to the codon immediately before the stop codon	This study

pJXW038	pAMD-BA-*lacZ* with *yncE* upstream sequence from −80 to +24 (relative to translation start site) and constitutive promoter from pAMD001	This study
pJXW039	pAMD-BA-*lacZ* with *cirA* upstream sequence from −173 to +24 (relative to translation start site) and constitutive promoter from pAMD001	This study
pJXW040	pAMD-BA-*lacZ* with *eptB* upstream sequence from −106 to +24 (relative to translation start site) and constitutive promoter from pAMD001	This study
pJXW041	pAMD-BA-*lacZ* with *sodB* upstream sequence from −55 to +24 (relative to translation start site) and constitutive promoter from pAMD001	This study
pJXW042	pAMD-BA-*lacZ* with *shiA* upstream sequence from −77 to +24 (relative to translation start site) and constitutive promoter from pAMD001	This study
Transcriptional fusions to *lacZ*		
pAMD033	pAMD-BA-*lacZ* with the sequence from -200 to +10 relative to the transcription start site of an antisense RNA found opposite *yaiP* (genome coordinate of the transcription start site is 382126 using the U00096.2 genome sequence) fused transcriptionally to *lacZ*, with a sequence immediately upstream of *lacZ* that includes an artificial Shine-Dalgarno sequence	(39)
pJXW037	pAMD033 with *dhaK* sequence from −300 (relative to translation start site) to the stop codon	This study
pJXW038	pAMD033 with *fepB* sequence from −300 (relative to translation start site) to the stop codon	This study
pJXW039	pAMD033 with *yegD* sequence from −300 (relative to translation start site) to the stop codon	This study
pJXW040	pAMD033 with *eptB* sequence from −300 (relative to translation start site) to the stop codon	This study
Translational fusions to *lacZ* with mutations		
pJXW042	pJXW023 with mutation in the *cirA* 5’ UTR	This study
pJXW043	pJXW023 with mutation in the *fepB* 5’ UTR	This study

Table S2. Oligonucleotides used in this study

JW2364	/5Phos/GATCGTCGGACTGTAGAACTCTGAACCTGTCGGTGGTCGCCGTATCATT/iSp18/CACTCA/iSp18/CAAGCAGAAGACGGCATACGATTTTTTTTTTTTTTTTTTTVN	Reverse transcription primer (60)
JW2365	AATGATACGGCGACCACCGA	Library primers (60)
JW2366	CAAGCAGAAGACGGCATACGA	Library primers (60)
JW2808	5Biosg/TCATCTCCGGGGGTAGAGCACTGTTTCG	rRNA removal (26)
JW2809	5Biosg/GGCTAAACCATGCACCGAAGCTGCGGCAG	rRNA removal (26)
JW2810	5Biosg/AAGGCTGAGGCGTGATGACGAGGCACT	rRNA removal (26)
JW2811	5Biosg/CGGTGCTGAAGCAACAAATGCCCTGCTT	rRNA removal (26)
JW3150	AGACCCTCGCGGAGA	RyhB qRT-PCR
JW3151	ACCCGGCTGGCTAAGTA	RyhB qRT-PCR
JW3379	GGTTCCTCATTACCGTTATCATATG	Artificial promoter *lacZ* fusion
JW3415	GGACGGATCCTCGAGCATGCTAGACAGCTGCATGCATCTTTG	Artificial promoter *lacZ* fusion
JW4984	AAAGTGTTGGACAAGTGCGAATGAGAATGATTATTATTGTCTCTAGACAGCTGCATGCAT	*ryhB* deletion
JW4985	ACACAAGCACTCCCGTGGATAAATTGAGAACGAAAGATCAAAAAGTGTAGGCTGGAGCTG	*ryhB* deletion
JW4986	CTGGGCGTGAATCTGCCTGA	*ryhB* mutation
JW4987	GGCAGATGGTAACGGGTGGT	*ryhB* mutation
JW5066	CATATGATAACGGTAATGAGGAACCCCCACGACAACCATAAATTC	Artificial promoter *lacZ* fusion of *ynfF*
JW5072	GGACGGATCCTCGAGCATGCGTACTCAGGGACGCGGCACA	Natural promoter *lacZ* fusion of *sodB*
JW5073	GGCCAGTGCCAAGCTTGCTAGTGCAGGTAATTCGAATG	*lacZ* fusion of *sodB*
JW5074	GGACGGATCCTCGAGCATGCGGTCGAGGGAAGAATTCCAC	Natural promoter *lacZ* fusion of *ykgJ*
JW5075	GGCCAGTGCCAAGCTTGCCATGCATGGATTCAGATTGC	*lacZ* fusion of *ykgJ*
JW5076	GGACGGATCCTCGAGCATGCGTCGTATTGAAGTACCAGAA	Natural promoter *lacZ* fusion of *dmsA*
JW5077	GGCCAGTGCCAAGCTTGCCGCATCAGGGATTTTCGTTT	*lacZ* fusion of *dmsA*
JW5079	GGCCAGTGCCAAGCTTGCGTAGATAAAGGGTTTGTTTG	*lacZ* fusion of *fumB*
JW5080	GGACGGATCCTCGAGCATGCTCACGCAAAAGTAGAATTGT	Natural promoter *lacZ* fusion of *nrfA*
JW5081	GGCCAGTGCCAAGCTTGCTGCGTTTATTTTTATCCTTG	*lacZ* fusion of *nrfA*

JW5082	GGACGGATCCTCGAGCATGCTATTGCATCGCT CCCAGGAG	Natural promoter *lacZ* fusion of *dhaK*
JW5083	GGCCAGTGCCAAGCTTGCCACATCATTGATCA ATTTTT	*lacZ* fusion of *dhaK*
JW5084	GGACGGATCCTCGAGCATGCACGCGCACTATG TCAACTCT	Natural promoter *lacZ* fusion of *fepB*
JW5085	GGCCAGTGCCAAGCTTGCGCGGTAGAGCGGGG CGAGTC	*lacZ* fusion of *fepB*
JW5086	GGACGGATCCTCGAGCATGCGAGCACAAAATG CTGCCTCG	Natural promoter *lacZ* fusion of *katG*
JW5087	GGCCAGTGCCAAGCTTGGATGGATATCGTCTG ACGTGC	*lacZ* fusion of *katG*
JW5088	GGACGGATCCTCGAGCATGCGTATGCATCTTT GACACATC	Natural promoter *lacZ* fusion of *napA*
JW5089	GGCCAGTGCCAAGCTTGCAAAGCTACGACGAC TGAGTT	*lacZ* fusion of *napA*
JW5090	GGACGGATCCTCGAGCATGCCCCTACTGGTTA TAATGCAA	Natural promoter *lacZ* fusion of *shiA*
JW5091	GGCCAGTGCCAAGCTTGCAGTGGAGATGAGCG TGGAGT	*lacZ* fusion of *shiA*
JW5092	GGACGGATCCTCGAGCATGCTCTTGTCTGATTT CGATGTT	Natural promoter *lacZ* fusion of *garP*
JW5093	GGCCAGTGCCAAGCTTGCTTCGTCAACGGTGT CCAGAA	*lacZ* fusion of *garP*
JW5094	GGACGGATCCTCGAGCATGCTTCCTTAATACTG ATAACTT	Natural promoter *lacZ* fusion of *cspB*
JW5095	GGCCAGTGCCAAGCTTGCTAAACCAGTCATTTT ATTTG	*lacZ* fusion of *cspB*
JW5096	GGACGGATCCTCGAGCATGCTGGCAATATGTT GAATTTGC	Natural promoter *lacZ* fusion of *ugpB*
JW5097	GGCCAGTGCCAAGCTTGCAGCTGTATAATGTA ACGGTT	*lacZ* fusion of *ugpB*
JW5098	GGACGGATCCTCGAGCATGCGGACAAGCAGTG CGGGCAAA	Natural promoter *lacZ* fusion of *yncE*
JW5099	GGCCAGTGCCAAGCTTGCTGAAAACAGATGAC GTAAAT	*lacZ* fusion of *yncE*
JW5100	GGACGGATCCTCGAGCATGCCGGCTATAT CCGGAATAGC	Natural promoter *lacZ* fusion of *yegD*
JW5101	GGCCAGTGCCAAGCTTGCACCGTAATCAAAAC CAATAA	*lacZ* fusion of *yegD*
JW5102	GGACGGATCCTCGAGCATGCGAAATAAGTT TCCTCCCTTC	Natural promoter *lacZ* fusion of *cirA*
JW5103	GGCCAGTGCCAAGCTTGCTACGAAAGGGTTCA ACCTAA	*lacZ* fusion of *cirA*
JW5104	GGACGGATCCTCGAGCATGCTGAGCATTACCA ATCACATC	Natural promoter *lacZ* fusion of *suhB*

JW5105	GGCCAGTGCCAAGCTTGCGGCGATGTTCAGCATCGGAT	*lacZ* fusion of *suhB*
JW5106	GGACGGATCCTCGAGCATGCGACAAACAAATTCCTTACTG	Natural promoter *lacZ* fusion of *cspG*
JW5107	GGCCAGTGCCAAGCTTGCTAAACCAGTCATTTTATTAG	*lacZ* fusion of *cspG*
JW5108	GGACGGATCCTCGAGCATGCCTGGTCATGTTGGAGTAGCA	Natural promoter *lacZ* fusion of *dppA*
JW5109	GGCCAGTGCCAAGCTTGCTGACTTTTTCAAGGAAATAC	*lacZ* fusion of *dppA*
JW5110	GGACGGATCCTCGAGCATGCTTCAGCTTCCGTGTGCATCA	Natural promoter *lacZ* fusion of *eptB*
JW5111	GGCCAGTGCCAAGCTTGCTGTAATCGATTTGATGTATC	*lacZ* fusion of *eptB*
JW5112	GGACGGATCCTCGAGCATGCGTCAGCGTTAACCCATATGA	Natural promoter *lacZ* fusion of *rnd*
JW5113	GGCCAGTGCCAAGCTTGCCGTGGTAATCATTTGGTAAT	*lacZ* fusion of *rnd*
JW5114	GGACGGATCCTCGAGCATGCGTAACGACAATGAACTCGGG	Natural promoter *lacZ* fusion of garD
JW5115	GGCCAGTGCCAAGCTTGCTTGTCTGATTTCGATGTTGG	*lacZ* fusion of garD
JW5116	GGCCAGTGCCAAGCTTGCCGCCTCTGTGGTATGGATTT	*lacZ* fusion of *ynfF*
JW 5119	CATATGATAACGGTAATGAGGAACCATGCACTTTGCGTGCCGCCC	Artificial promoter *lacZ* fusion of *ynfF*
JW5170	TTCATGCATTGCTAGCTTATTTACCCCAGTTAAGGG	Transcriptional lacZ fusion of *dhaK*
JW5171	TTCATGCATTGCTAGCTTAAAACAGCGCCTTAAGCC	Transcriptional lacZ fusion of *fepB*
JW5172	TTCATGCATTGCTAGCTTAACGAAACACCACTTCCG	Transcriptional lacZ fusion of *yegD*
JW5173	TTCATGCATTGCTAGCTTAGTTAGCCGCTGCCTCTT	Transcriptional lacZ fusion of *eptB*
JW5175	GGCCAGTGCCAAGCTTGCCAGCAGGTCGAAACGGTCGA	Natural promoter long-version *lacZ* fusion of *katG*
JW5367	TGAAAGCACGACATTGCTATACTTGCTTCCAGTATTACT	*ryhB* mutagenesis for *cirA*
JW5368	AGTAATACTGGAAGCAAGTATAGCAATGTCGTGCTTTCA	*ryhB* mutagenesis for *cirA*
JW5375	CGGCGAGTCGCTCCTGCCAC	*ryhB* mutagenesis
JW5376	CCGGCTTCGCATGGCGACGA	*ryhB* mutagenesis
JW5381	CAAGGGGACGTGAAGAAGAGTATAGCGATAACCCATTTTATT	*cirA* mutation

JW5382	AATAAAATGGGTTATCGCTATACTCTTCTTCAC GTCCCCTTG	*cirA* mutation
JW5707	CACGACATTGCTCACATTTTCTCCAGTATTACT TAGCCA	*ryhB* mutagenesis for *fepB*
JW5708	TGGCTAAGTAATACTGGAGAAAATGTGAGCAA TGTCGTG	*ryhB* mutagenesis for *fepB*
JW5711	ACCTTATTAATAACAGGAGAATGATTTGTGAG ACTCGCC	*fepB* mutation
JW5712	GGCGAGTCTCACAAATCATTCTCCTGTTATTAA TAAGGT	*fepB* mutation
JW6545	CATATGATAACGGTAATGAGGAACCCGAGCAA TGTCATGACAGTG	Artificial promoter *lacZ* fusion of *nrfF*
JW6546	CATATGATAACGGTAATGAGGAACCTAATTTT CTTTTCCCTTGCC	Artificial promoter *lacZ* fusion of *dhaK*
JW6547	CATATGATAACGGTAATGAGGAACCATTATTG ATGGATTCGCATA	Artificial promoter *lacZ* fusion of *fepB*
JW6548	CATATGATAACGGTAATGAGGAACCACACTGT AGAGGGGAGCACA	Artificial promoter *lacZ* fusion of *katG*
JW6549	CATATGATAACGGTAATGAGGAACCATAACAA GAGCGTAACGATG	Artificial promoter *lacZ* fusion of *yncE*
JW6550	CATATGATAACGGTAATGAGGAACCATCGTTA CGC CGCAATCAAA	Artificial promoter *lacZ* fusion of *cirA*
JW6551	CATATGATAACGGTAATGAGGAACCGCGCGTG TAGATTTTACTTA	Artificial promoter *lacZ* fusion of *eptB*
JW6553	CATATGATAACGGTAATGAGGAACCATACGCA CAATAAGGCTATT	Artificial promoter *lacZ* fusion of *sodB*
JW6554	CATATGATAACGGTAATGAGGAACCGTTCGTT TATAGATCGACGG	Artificial promoter *lacZ* fusion of *shiA*
EM1689	GACTACAAAGACCATGACGGTGATTATAAAGA TCATGATATCGACTACAAAGATGACGACGATA AATAGTAA GTGTAGGCTGGAGCTGCT	FLAG-tagging *hfq*
EM1690	GGATCGCTGGCTCCCCGTGTAAAAAAACAGCC CGAAACCTTACATATGAATATCCTCCTTAG	FLAG-tagging *hfq*
EM1691	GCAGAATACTTCCGCGCAACAGGACAGCGAAG AAACCGAAGACTACAAAGACCATGACGG	FLAG-tagging *hfq*
